# Potential for Family Planning Program Sustainability Across 33 Countries in Two Regions of sub-Saharan Africa

**DOI:** 10.12688/gatesopenres.16371.1

**Published:** 2025-11-10

**Authors:** Karen Hardee, Rebecca Rosenberg, Imelda Zosa-Feranil

**Affiliations:** 1 Hardee Associates LLC, Arlington, VA, 22207, USA; 2 Avenir Health, Glastonbury, CT, USA; 3 Avenir Heah, Glastonbury, CT, USA

**Keywords:** Family planning, sustainability, national composite index of family planning, NCIFP, sub-Saharan Africa

## Abstract

**Background:**

Sustainability, with countries taking ownership for funding and ensuring access to services and contraceptive commodities, has long been an aim for FP. Recent shocks to donor funding have added urgency to country sustainability. Findings from the 2023 National Composite Index of Family Planning (NCIFP) and its special questions on Sustainability provide a broad view from 33 counties across two sub-regions of sub-Saharan Africa of expert respondents’ perceptions in that year on the potential for program sustainability.

**Methods:**

The cross-national NCIFP survey has been conducted since 2014. Its 2023 special questions were designed to take the pulse of stakeholders steeped in their countries’ FP program about the state of sustainability, actions being taken, and main challenges their countries faced. Items related to donor dependency, coordination, FP in country plans for Universal Health Coverage (UHC), planning for future demand, and key barriers to sustainability.

**Results:**

Both regions scored highest on the extent to which FP is included in UHC plans and lowest on the items related to donor reliance, indicating high dependency for contraceptive commodities and programmatic support. Donor dependence and lack of domestic financing were most often cited barriers, with human resources; commodities and logistics; and sociocultural, gender and religious barriers all receiving over 10% of the responses on key challenges. The extent to which governments had developed plans to make their national FP programs more sustainable to meet demand varied.

**Conclusion:**

These data provide an important snapshot of family planning programs across SSA prior to the abrupt cancellation of USAID funding in January 2025. Country context, including national wealth and government effectiveness, is important. From the 2023 NCIFP, understanding program experts’ perspectives, and identifying similar and unique challenges countries face, is critical to help shape support to strengthen the capacity of countries to move towards FP program sustainability.

## Background/Introduction

Sustainability, with countries taking responsibility for ensuring access to services and contraceptive commodities, has long been an aim for family planning (FP) programs, with increasing calls for government ownership, and localization along with domestic resource mobilization. Attention to sustainability in FP programming has focused on designing interventions to be sustainable ( World Health Organization/ExpandNet, 2011), maintaining interventions when funding ends ( Speizer et al., 2019), sustaining interventions and organizations ( Ashford and Haws, 1992), and sustaining broader programs ( Stephenson et al., 2004; Khalifa et al., 2001; USAID, 1999; Shediac-Rizkallah and Bone, 1998), including through scaling up high impact practices ( Hardee et al., 2017), programming and strengthening health systems ( Finkle et al., 2024), and through graduating countries from donor assistance ( Cromer et al., 2004; Chaudhury, et al. 2012; Do et al., 2024). More recent attention has focused on promoting the inclusion of FP into universal health coverage (UHC) ( Doraiswamy, 2021; Holtz and Sarker, 2018; FP2030 et al., 2022). The early 2025 abrupt cancellation of USAID funding by the Trump administration ( https://www.independent.co.uk/news/world/usaid-global-health-hiv-women-africa-b2694023.html), adds critical importance to the topic of sustainability.

### Defining program sustainability

The issue of FP program sustainability conjures up concerns over resource mobilization, often thought of as mobilizing financial resources. For decades, external assistance from developed countries has been a major source of funding for national FP programs in developing countries. The estimated cost of ending the unmet need for FP in 120 priority countries (countries with the majority of global unmet need) from 2020 to 2030 is $68.5 billion, yet pledges for development assistance for this decade in those countries is only $8.6 billion, leaving a $59.9 billion funding gap ( UNFPA, 2020). Promoting domestic financing, or governments taking on a larger share of funding for FP programs – both contraceptives and services – remains an urgent call ( UNFPA, 2024; HIPs, 2018), particularly with the abrupt closure of USAID in early 2025 by the Trump Administration, halting family planning assistance from the United States.

Program sustainability, however, is a much broader issue than domestic and international funding. Sustainability refers to the ability of a program to continue producing positive outcomes and impacts over time ( Schell et al., 2013). This implies that programs should be designed, supported, and implemented with long-term impact in mind, ensuring that they have the necessary resources, support, and capacity to continue producing positive outcomes and impacts.

The national family planning program and its public, private and NGO institutions can provide current and potential clients with the information and services necessary to obtain the benefits of quality family planning on a continuous basis without external aid.

There is no one definition of sustainability in FP, given varying focus on what is to be sustained (a practice, an intervention, an organization, or a broader program), and programs are operating in a rapidly changing landscape, with greater attention to country ownership and localization, and reassessing outcomes of success. One useful definition of broader FP program sustainability comes from a USAID-funded population project in Egypt: “The national family planning program and its public, private and NGO institutions can provide current and potential clients with the information and services necessary to obtain the benefits of quality family planning on a continuous basis without external aid” ( Khalifa et al., 2001), likely guided by a similar definition of program sustainability from the USAID Africa Bureau Office of Sustainable Development ( USAID, 1999). Similarly, in their 2012 review of USAID’s experience in graduating and phasing-out health programs, with most examples coming from graduation of FP assistance, Chaudhury et al. (2012: 3) defined sustainability as “the capacity of a host country entity to achieve long-term success and stability and serve its population without interruption and without reducing the quality of services after external assistance ends.” All of these definitions assume time for planning for sustainability.

### Components of program sustainability

In order for voluntary family planning programs to be successful and sustainable, there needs to be strong national capacity to implement and manage programs, including capacity to mobilize and spend the necessary financial resources for family planning commodities, service delivery, demand creation, and training.

Experience with programs around the world has generated lists of key components of FP programs to consider for sustainability ( Khalifa et al., 2001; Cromer et al., 2004; Santiso-Gálvez and Bertrand, 2007; Chaudhry et al., 2012; HIPs, 2018; Speizer et al., 2019). The Family Planning High Impact Practices (HIPs) Partnership, which groups its high impact practices into those related to the enabling environment, service delivery, and social behavior change, notes that “in order for voluntary family planning programs to be successful and sustainable, there needs to be strong national capacity to implement and manage programs, including capacity to mobilize and spend the necessary financial resources for family planning commodities, service delivery, demand creation, and training” ( HIPs, 2018).

In their review of graduation from USAID assistance for health programming, with most examples coming from FP programs, Chaudhry et al. (2012) identified four key pillars for increasing the sustainability or self-sufficiency of health programs: (1) country-led financing; (2) major policy and regulatory reforms that ensure a strong enabling environment; (3) institutional strengthening of the government, NGO, private, and civil society sectors; and (4) leadership and stewardship with committed leaders managing resources sustainably. Khalifa et al. (2001) similarly noted the importance of a strong enabling environment, including financial sustainability even without external assistance, and institutional sustainability to ensure the capacity to provide ongoing quality services and contraceptives. They added an additional factor, namely demand sustainability - the ability of the program to meet current and growing demand for services.

Related elements, adapted and expanded from Santiso-Gálves and Bertrand (2007) and others, encompass understanding the sociocultural context as it relates to FP. These elements include integrating FP with other health services to better meet clients’ needs and increase the efficiency of service delivery; enabling the ministry of health (or other relevant ministry/department) to accept its rightful responsibilities of providing rights-based and high quality FP services; working with other ministries, (e.g., education, labor, economy, and finance ministries); improving coordination among agencies working in FP; ensuring adequate trained and supervised staffing and service points, including community-based; and supporting monitoring and evaluation of the program. An evaluation of programs in four countries (Morocco, Indonesia, Peru, and Honduras) that had sustained the transition from USAID financial support – with planning prior to the transitions – found that leadership, commitment, and coordination between multiple sectors were considered “critical for a successful and sustainable transition of FP programs” ( Do et al., 2024: 9).

Assessing FP programming with a sustainability lens can provide valuable insight into the potential of programs in sub-Saharan Africa to become, and remain, sustainable, and the challenges facing countries as they navigate the donor landscape that has abruptly shifted in 2025.

## Purpose

This paper uses a unique dataset from a cross-national survey in 2023 designed to take the pulse of stakeholders steeped in their countries’ FP program about the state of sustainability of the program as of that year, actions being taken, and the key challenges their countries faced. The paper focuses on two sub-regions of sub-Saharan Africa, namely 17 countries in East and Southern Africa (ESA), and 16 countries in West and Central Africa (WCA) that took part in the 2023 round of the National Composite Index of Family Planning (NCIFP), described in more detail in the methodology section. We focus on sub-Saharan Africa because many country programs in this region have been dependent on donor funding which had already been decreasing by 2023, prior to the funding cut shock in 2025, so many of these countries may be facing challenges with domestic financing and other aspects of sustainable programs. Understanding the state of program sustainability from the perspective of program experts, and both similar and unique challenges faced by countries, will help shape the support needed to strengthen the capacity of countries to move towards sustainability.

Common elements emerge from the various definitions and elements of sustainability identified in the literature. For purposes of this paper, we focus on key elements identified by past studies that encompass an *enabling environment* that includes financial resources, political and policy support, and coordination; *service delivery* capacity of the program to provide and monitor high-quality services to meet ongoing demand; with *attention to the socio-cultural context* of the country. The paper also addresses country efforts to plan for sustainability – as reflected in 2023.

## Methods

Building on the Family Planning Effort (FPE) score, measured over several decades of FP programming starting in the 1970s ( Lapham & Mauldin, 1984; Mauldin & Ross, 1991; Ross & Stover, 2001; Kuang & Brodsky, 2016), the NCIFP was developed to support measurement efforts by the global initiative Family Planning 2020 (FP2020, now FP2030) to capture indicators related to an enabling policy environment and a rights-based approach to FP services – important program dimensions for which information is not easily captured by service statistics or population-based surveys. The NCIFP, fielded in 2014, 2017, 2021 and 2023, focuses on FP policies, plans and structures, including data systems, that pertain to quality of care, choice, accountability, and equity. The 2023 NCIFP Global Report ( Rosenberg et al., 2024) presents the main findings of the 2023 study and compares the 2023 findings with the 2017 and 2021 results to illustrate change over time.

The NCIFP uses a key informant approach, identifying experts in each country who have a comprehensive understanding of the FP program. Data collection at the country-level is managed by a Track20 Monitoring and Evaluation Officer (M&E Officer) or a local consultant who is familiar with the national FP program and could identify people who could gauge the effort levels of its various features. Study leaders of the 2023 NCIFP contacted countries that participated in the 2021 NCIFP, the 2017 NCIFP, the 2014 FPE/NCIFP survey and earlier FPE data collection to identify country consultants. The consultant in each country instructed 12-15 local respondents in questionnaire completion and followed up to obtain the responses. Participants included individuals who were considered FP program leaders, experts, and observers. To obtain a variety of perspectives, respondents worked in four different capacities: inside the FP program, in government but outside the FP program (e.g. Parliamentarians), in local civil society organizations (CSOs), and non-governmental organizations (NGOs) and private entities, in local academic or research organizations, and resident staff of international agencies.

The 2023 questionnaire was conducted via a user-friendly online form linked to an online cloud to enable study leaders to conduct real-time data quality checks and rapid follow-up. Each respondent was given a unique ID number that was kept secure, allowing only study leaders access to both the online responses and respondent identifiers. Identifying information was used for follow-up purposes only and is not present in any of the analyses.

Data were exported to Excel, with checks for consistency and data quality. The responses from each expert in a country were averaged to obtain a country score for each individual question. The total score, and scores for each dimension were calculated from averaging across the individual questions. All questions were coded in the same direction, so that responses that positively support an enabling environment for FP correspond to higher scores and negative responses correspond to lower scores. For example, scoring for the item, “Extent to which the national family planning program is reliant on donor agencies for funding contraceptive commodities,” was reversed so that high donor dependency corresponds to lower scores and little or no donor dependency corresponds to high scores. Analytic techniques included graphical and correlation approaches.

Over time, the NCIFP incorporated a special topic dimension at the end of the standard NCIFP questionnaire (identified in the questionnaire as a supplement) in response to urgent global issues. The 2021 NCIFP special topic questions collected information on the impact of the COVID-19 pandemic on the national FP program ( Hardee et al., 2024). In 2023, special topic questions were designed to provide information on the sustainability of the national FP program. The questions on sustainability included items related to donor dependency, coordination, FP as part of country plans for UHC, and planning for future demand (please refer to the special topic questions on sustainability in the 2023 NCIFP questionnaire shown in Box 1).

Box 1. **Special questions on sustainability, 2023 NCIFP**

Special question (SQ) 1. Extent to which the national family planning program is reliant on donor agencies for funding each of the following: (1 = not at all reliant; 10 = extremely reliant)

*Note: For this question, you must rate each of the following areas separately.*

  Contraceptive commodities

  Program activities

SQ 2. Extent to which the government coordinates funding mechanisms across sources (national and local government funding, donor financing, in-kind contributions, etc.) (1 = not at all; 10 = highly coordinated)

SQ 3. Extent to which the health management information system (HMIS) supports timely and complete reporting of data to inform monitoring and planning (1 = not at all; 10 = extremely effective)

SQ 4. Extent to which the logistics management information system (LMIS) supports timely and complete reporting of data to inform commodity management (1 = not at all; 10 = extremely effective)

SQ 5. Extent to which family planning is part of the country’s plans for Universal Health Coverage (UHC) (1 = not at all; 10 = extremely important)

SQ 6. Extent to which the government has developed plans to make the national family planning program more sustainable to achieve each of the following (1 = not at all; 10 = extremely strong effort)

*Note: For this question, you must rate each of the following areas separately.*

  Reduced reliance on donors

  Realistic projections of commodities needed to meet demand

  Adequate recruitment of skilled staff to meet demand

  Adequate fielding of staff to meet demand

  Adequate infrastructure and equipment to meet demand

  Programmatic support from leaders at the subnational level

SQ 7. In your opinion, what are the *three* largest barriers to sustainability for the family planning program in your country?

In addition to the six close-ended questions about specific sustainability issues, respondents were also asked, “In your opinion, what are the three largest barriers to sustainability for the family planning program in your country? *”* to give key informants an opportunity to provide more information or qualify their responses with few constraints and in their own words ( Cibelli Hibben et al., 2024). While open-ended questions can generate non-response rates approaching 20 percent ( Asare-Marfo, 2021) *,* the non-response rate for this open-ended question was 12 percent across the regions (9% for ESA and 15% in WCA) when all three possible responses were combined (for a total of 1,323 possible responses). Of the 228 respondents in the 17 countries in ESA, only six (3%) did not provide a first response to the question, and in WCA among the 213 respondents from 16 countries, 22 (10%) did not provide a first response to the question. Among those who did respond, across the two regions only three respondents (1 in ESA and 2 in WCA) said their country faced no barriers to sustainability.

Responses for this question were translated as needed then analyzed qualitatively by tagging responses for each of the three barriers according to themes that emerged ( Box 2) and grouping these themes into the framework for this paper, namely enabling environment, program/service delivery; and attention to socio-cultural context. Many themes also relate to the main dimensions of the NCIFP, and the closed-ended questions on sustainability, which focused on the enabling environment and program/service delivery ( Box 1). The themes also align with the many components of sustainability articulated above, namely enabling environment (including financial sustainability), institutional sustainability; and demand sustainability ( Khalifa et al., 2001).

Box 2. **Barriers to FP Program Sustainability: Emergent Themes**

**Enabling environment**

  • Domestic financing; donor dependence

  • Political will; policy issues; accountability

  • Coordination; planning; competing interests

  • Private sector engagement

**Program/service delivery**

  • Commodities; supply chain

  • Human Resources; number and training

  • Infrastructure; coverage; access

  • Quality of care/services

  • Data; M&E

**Attention to socio-cultural context**

  • Sociocultural, gender, religious barriers

  • Insufficient attention to SBC

**Other**

**No response (NR)**

Given the sample size and high response rate, we present descriptive results from the open-ended question as percentages of possible responses (684 for ESA and 639 for WCA) that identified the various themes as key barriers to sustainability. Providing this information by country highlights the variations among countries in the barriers to sustainability. Additionally, we include quotes to illustrate the barriers identified to provide insights into unique as well as common problems affecting countries. These findings enhance the results from the close-ended questions on sustainability.

### Ethical approval and consent

Since the first round in 2014, the NCIFP has been conducted within a monitoring and evaluation framework focused on family planning programs, rather than under a research protocol. Still, written informed consent was obtained to take part in the NCIFP and all data has been anonymized.

## Results

### Overview

**Figure 1.**
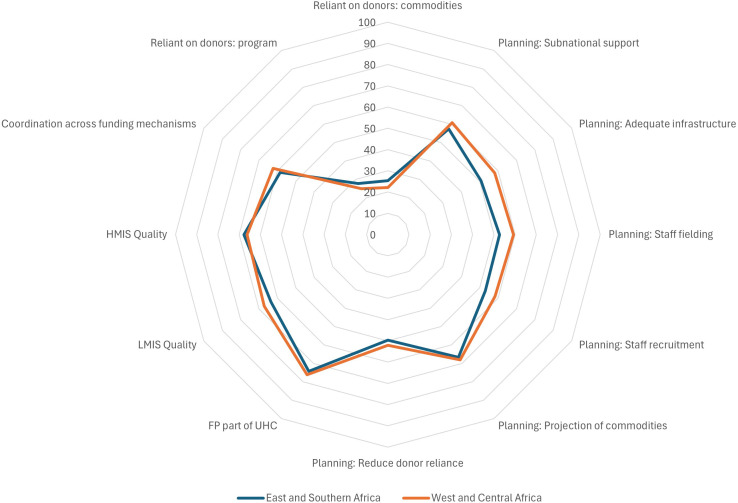
Sustainability Items, by SSA Region.

Figure 1 graphically represents the scores for each of the 12 items generated from the six close-ended sustainability questions, by region and Table 1 presents the numeric scores. The close-ended items are organized into the main themes identified in the literature review, namely *enabling environment* and *program/service delivery.* The theme of *attention to socio-cultural context* only emerged in the qualitative responses.

**Table 1.** Sustainability scores, by SSA Region.

Theme	Item	East and Southern Africa	West and Central Africa
Enabling Environment	Reliant on donors: commodities	25	22
	Reliant on donors: program	28	25
	Coordination across funding mechanisms	59	62
Program/service delivery	HMIS quality	68	66
	LMIS quality	64	67
	FP part of UHC	74	76
	Planning: Reduce donor reliance	50	52
	Planning: Projection of commodity needs	67	68
	Planning: Staff recruitment	53	58
	Planning: Staff fielding	53	59
	Planning: Adequate infrastructure	51	58
	Planning: Subnational support	57	61
	**Total score**	**54**	**56**

Both regions scored highest on the extent to which FP is included in plans for UHC and lowest on the items related to donor dependency, indicating that as of 2023, SSA governments are highly dependent on donors for contraceptive commodities and programmatic support.

Each of the items included in the special questions on sustainability represent selected components of the sustainability of an FP program, namely donor reliance; inclusion in UHC; coordination; data systems; and planning. To gain a better understanding of country performance, as described in Table 2, we generated scores for each of these components. For consistency, the components were grouped into the main themes of enabling environment and program/service provision in Table 2. ^1^ However, results are presented in components, rather than condensed into the broader main themes to show more of the nuance in the range of scores.

**Table 2.** Components of sustainability.

Theme	Component	Sustainability Items
**Enabling Environment**	**Donor Reliance**	Average of extent to which: - the national family planning program is reliant on donor agencies for funding contraceptive commodities - the national family planning program is reliant on donor agencies for funding program activities
	**Coordination**	Extent to which the government coordinates funding mechanisms across sources (national and local government funding, donor financing, in-kind contributions, etc.)
**Program/service delivery**	**Data Systems**	Average of extent to which: - the health management information system (HMIS) supports timely and complete reporting of data to inform monitoring and planning - the logistics management information system (LMIS) supports timely and complete reporting of data to inform commodity management
	**Universal Health Coverage**	Extent to which family planning is part of the country’s plans for Universal Health Care (UHC)
	**Planning**	Average of: extent to which the government has developed plans to make the national family planning program more sustainable to achieve: - reduced reliance on donors - realistic projections of commodities needed to meet demand - adequate recruitment of skilled staff to meet demand - adequate fielding of staff to meet demand - adequate infrastructure and equipment to meet demand - programmatic support from leaders at the subnational level

**Figure 2.**
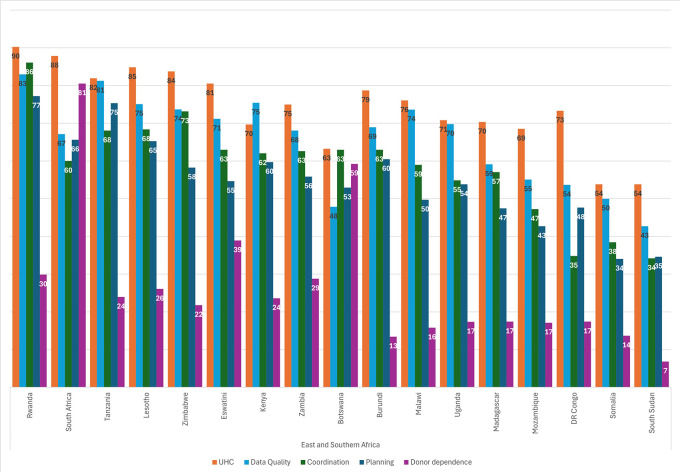
Sustainability component scores, by country in ESA.

Figure 2 shows the scores for each of the five sustainability components for the countries in ESA. As was evident from the regional scores, every country except South Africa and Botswana scored lowest for Donor Reliance, and UHC was the highest scoring component in every country except Kenya. Countries generally scored high for Data Systems (HMIS and LMIS), with the exception of Botswana, where this component lagged behind. Planning was the second-lowest scoring component for many ESA countries, indicating some difficulty in keeping an eye toward the future and anticipating future needs.

**Figure 3.**
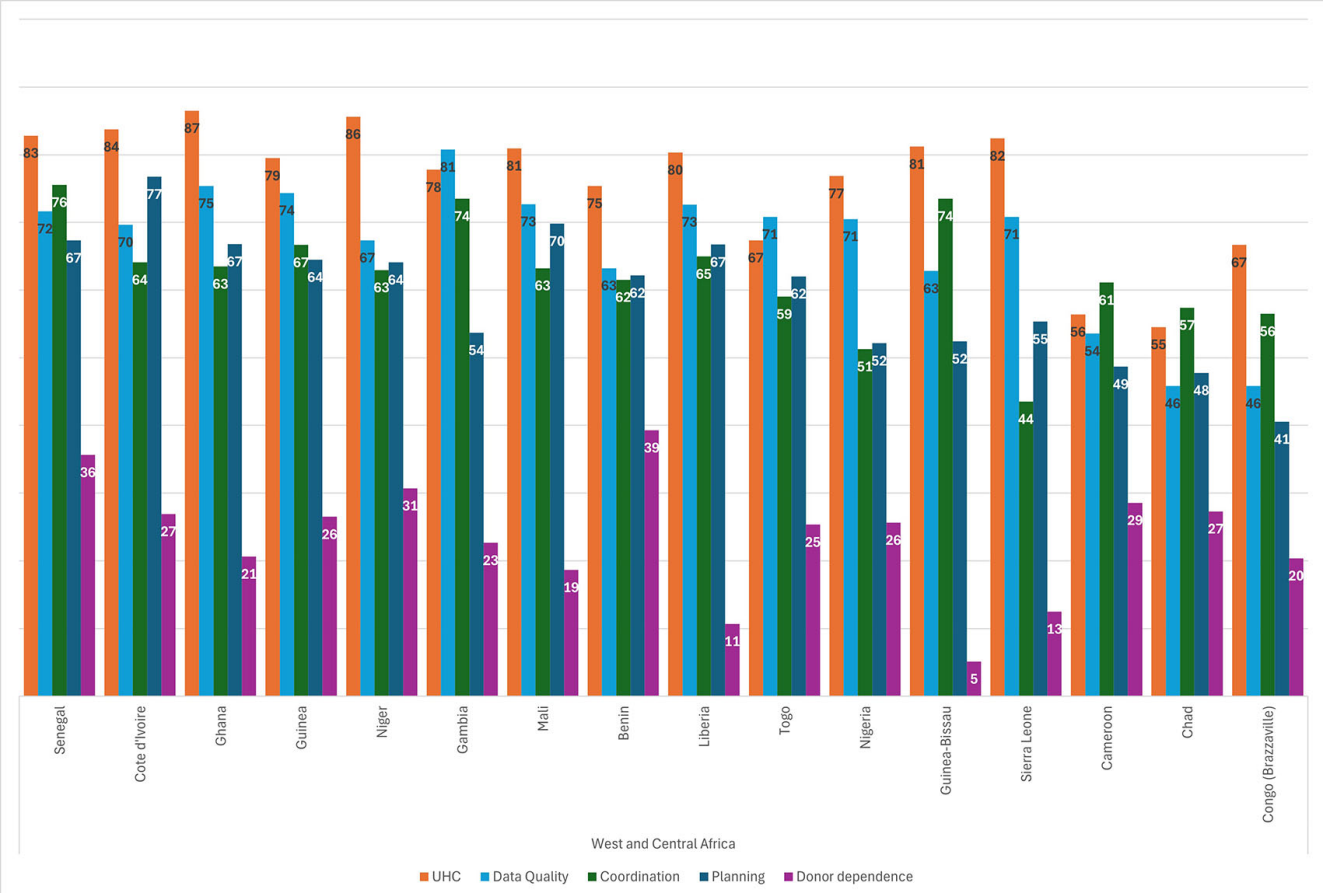
Sustainability component scores, by country in WCA.

Figure 3 shows the scores for each sustainability component for the countries in WCA. Every country scored lowest for donor reliance, indicating a high degree of donor dependency across the region. As in the ESA region, most countries in WCA scored highest for UHC, with the exception of Cameroon, Chad, Gambia and Togo. Scores for the remaining three categories were mixed. The second-lowest component was Planning for five countries (Cameroon, Congo, Gambia, Guinea, and Senegal), and Coordination was the second-lowest scoring for nine countries. Chad was the only country to score second-lowest for Data Systems.

Compared to the other sustainability components, donor reliance was the lowest-scoring sustainability component for almost every country, indicating a common challenge facing most countries in SSA. Table 3 shows the donor reliance scores alongside scores for plans to achieve reduced donor reliance for each country, by region. Scores for donor reliance in ESA ranged from 81 in South Africa (indicating very low levels of donor reliance), to 7 in South Sudan (indicating very high levels of donor reliance). In WCA, scores ranged from 39 in Benin to 5 in Guinea-Bissau.

**Table 3.** Donor Reliance and plans to achieve reduced donor reliance, by region and country.

Region	Country	Donor Reliance (low score indicates high donor reliance)	Plans to achieve reduced donor reliance
East and Southern Africa	South Africa	81	72
	Botswana	59	54
	Eswatini	39	53
	Rwanda	30	68
	Zambia	29	48
	Lesotho	26	60
	Tanzania	24	73
	Kenya	24	58
	Zimbabwe	22	61
	DR Congo	17	39
	Madagascar	17	44
	Uganda	17	49
	Mozambique	17	19
	Malawi	16	45
	Somalia	14	28
	Burundi	13	50
	South Sudan	7	22
West and Central Africa	Benin	39	53
	Senegal	36	62
	Niger	31	58
	Cameroon	29	50
	Chad	27	53
	Cote d'Ivoire	27	65
	Guinea	26	60
	Nigeria	26	47
	Togo	25	61
	Gambia	23	40
	Ghana	21	58
	Congo	20	44
	Mali	19	61
	Sierra Leone	13	47
	Liberia	11	50
	Guinea-Bissau	5	24

Despite recognition of the challenge of donor dependence, are countries planning to address the challenge of financing? For example, Tanzania scored 24 on donor reliance, indicating a fairly high level of dependency, but scored 73 for the extent to which the government has developed plans to achieve reduced reliance on donors. Mozambique, on the other hand scored low on both items, indicating a heavy reliance on donors (17 points) and a lack of plans for reducing this dependency (19 points).

**Figure 4.**
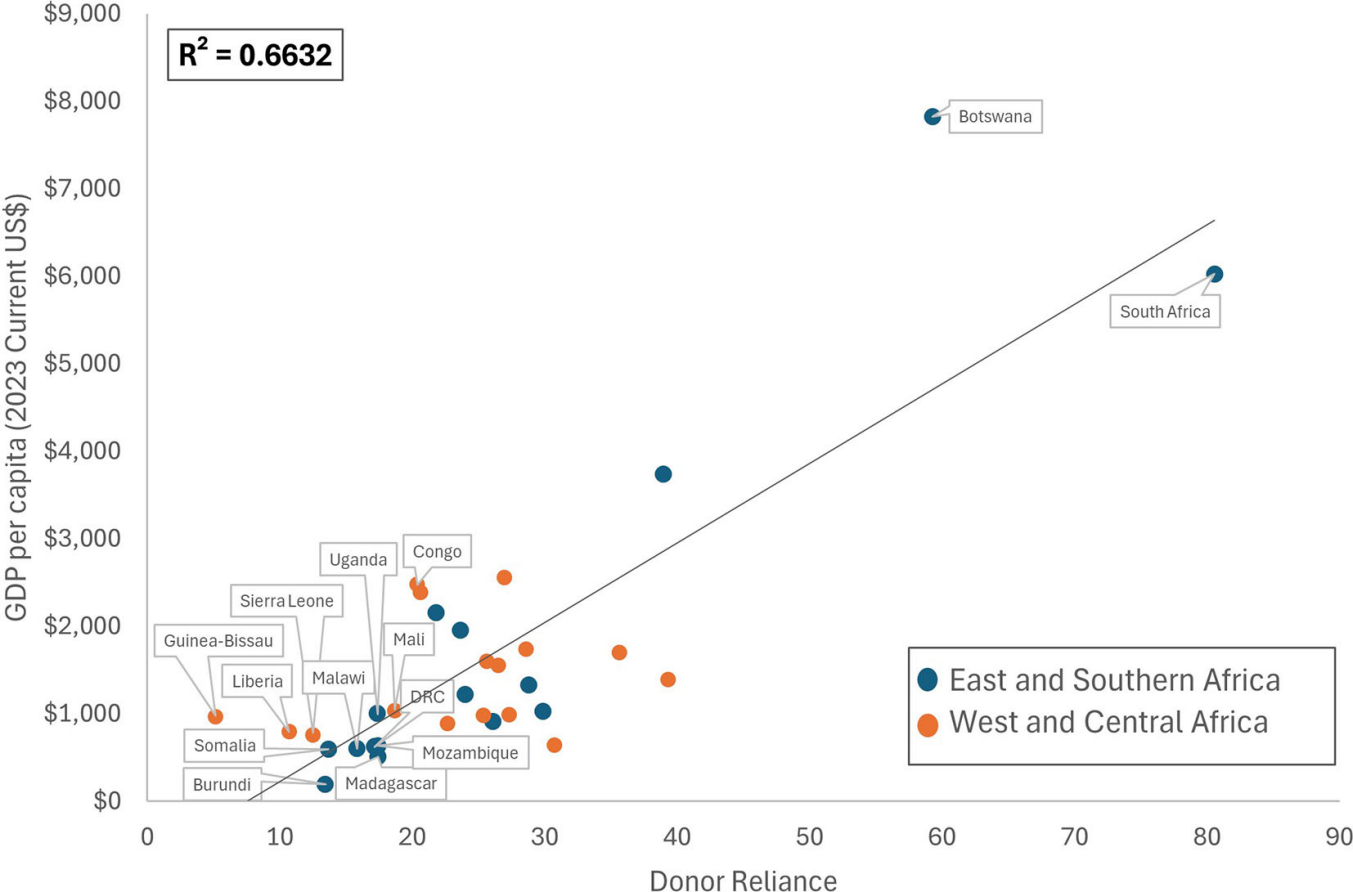
Donor reliance scores and GDP per capita (current $US).

Country context is an important element of how reliance on donor support will impact sustainability of the FP program. For example, countries with limited fiscal space are more likely to experience programmatic collapse following donor withdrawal compared to countries with a higher degree of national wealth. Figure 4 shows the relationship between World Bank data on GDP per capita ( https://data.worldbank.org/indicator/NY.GDP.PCAP.CD) and donor reliance. With few exceptions, the 12 countries with a donor reliance score of 20 or below ^2^ (indicating a strong reliance on donor funding for contraceptive commodities and programmatic activities) also had the lowest GPD per capita. The FP programs in these countries are particularly vulnerable to changes in donor funding and are therefore the least financially sustainable.

## Key barriers to sustainability of the family planning program

As a final, open-ended question on sustainability, respondents were asked to give their top three barriers to sustainability. As noted in the methodology section, the non-response rate was low (across both regions only 6% of respondents did not list at least one challenge) and very few NCIFP respondents said their country faced no barriers; in fact, respondents had a lot to say about barriers to sustainability ( Box 2 in the methodology section lists the themes that emerged). As shown in Table 4 **,** the barrier most often identified by respondents in both ESA and WCA was, not surprisingly, the dependence on donors for funding and lack of domestic financing. Among all 1,323 possible responses across all three responses, 24 percent related to financing, including for commodities. This seemingly low percentage obscures the finding that financing was the first barrier mentioned by around half of the respondents in both regions (not shown). These percentages also reinforce that respondents noted a range of barriers in addition to funding.

**Table 4.** Top 3 barriers to sustainability * identified by respondents in the 2023 NCIFP, combined results by SSA region.

Programming theme	Identified barrier	ESA (17 countries)	WCA (16 countries)	Average across regions
**Enabling environment**	**Domestic financing; donor dependence**	23%	24%	24%
	**Political will; policy issues; accountability**	8%	6%	7%
	**Coordination; planning; competing interests**	2%	4%	3%
	**Private sector engagement**	2%	<1%	1%
**Program/Service delivery**	**Human Resources; number and training**	15%	11%	13%
	**Commodities; supply chain**	9%	12%	11%
	**Infrastructure; coverage; access**	8%	6%	7%
	**Quality of care/services**	3%	3%	3%
	**Data; M&E**	2%	3%	3%
**Attention to socio-cultural context**	**Sociocultural, gender, religious barriers**	10%	8%	9%
	**Insufficient attention to SBC**	5%	5%	5%
	**Other**	3%	<1%	2%
	**NR**	9%	15%	12%
**Total number of responses (each country's number of respondents x 3)**		684	639	1,323

* The percentages represent the responses for the corresponding barrier as a percentage of all barriers. No response (NR) is included in the denominator because some respondents did not list all three barriers.

‘Human resources, number and training,’ under program/service delivery barriers, was noted in 13 percent on average of the possible three responses under ‘program/service delivery’ barriers (15% in ESA and 11% in WCA). Also under program/service delivery barriers, commodity/supply chain issues were noted in 11% of responses across the regions. It is instructive to consider resources and commodities together since most responses about funding in the open-ended question did not differentiate between funding for programming versus commodities.

In response to the open-ended question, respondents also highlighted the social dimensions of barriers to sustainability. Ten percent of respondents across the regions identified barriers to sustainability related to sociocultural and gender norms and practices and to religious objections, with an additional five percent calling out insufficient attention to social behavior change (SBC) by their countries’ FP programs.

Table 5 takes a closer look at respondents’ perceptions of barriers at the country level and underscores both common barriers and differences across counties in the prioritization of barriers. The percentages of responses within countries give a snapshot of what barriers are considered most pressing.

**Table 5.** Top 3 barriers to sustainability * identified by respondents in the 2023 NCIFP, combined results by country and SSA region.

		Enabling environment				Program/Service delivery					Attention to socio-cultural context		Other	NR
Region and country	Total number of responses **	Domestic financing; donor dependence	Political will; policy issues; accountability	Coordination; planning; competing interests	Private sector engagement	Human Resources; number and training	Commodities; supply chain	Infrastructure; coverage; access	Quality of care/services	Data; M&E	Sociocultural, gender, religious barriers	Insufficient attention to SBC		
**East and Southern Africa**														
Botswana	39	**18%**	8%	5%	0%	**15%**	**15%**	5%	8%	8%	0%	5%	0%	13%
Burundi	36	**33%**	3%	3%	0%	**22%**	3%	3%	3%	0%	**25%**	3%	0%	3%
DR Congo	45	**36%**	4%	4%	2%	**16%**	**11%**	9%	2%	2%	**11%**	2%	0%	0%
Eswatini	36	**17%**	8%	6%	0%	**11%**	**22%**	0%	6%	3%	**11%**	3%	0%	14%
Kenya	36	**36%**	**19%**	0%	0%	**17%**	6%	8%	3%	0%	8%	0%		3%
Lesotho	39	**15%**	3%	3%	5%	**13%**	8%	**15%**	5%	3%	8%	**10%**	0%	13%
Madagascar	45	**16%**	**16%**	2%	4%	**13%**	**11%**	**16%**	0%	0%	2%	2%	0%	18%
Malawi	39	**26%**	8%	0%	0%	**10%**	3%	5%	3%	0%	**13%**	**13%**	5%	15%
Mozambique	36	**31%**	6%	6%	6%	6%	**17%**	0%	0%	3%	6%	0%	6%	17%
Rwanda	48	**10%**	0%	0%	2%	**19%**	8%	**17%**	2%	0%	**25%**	2%	8%	6%
Somalia	39	**21%**	**10%**	5%	0%	5%	**10%**	**10%**	3%	0%	**15%**	**15%**	5%	0%
South Africa	36	**11%**	8%	3%	0%	**28%**	**14%**	**11%**	8%	0%	3%	0%	3%	11%
South Sudan	39	**26%**	**10%**	0%	0%	**10%**	3%	**10%**	0%	3%	**21%**	8%	**10%**	0%
Tanzania	48	**19%**	6%	2%	0%	6%	6%	6%	8%	2%	8%	**15%**	4%	17%
Uganda	48	**29%**	**19%**	4%	2%	**13%**	6%	4%	8%	0%	8%	2%	0%	4%
Zambia	36	**28%**	6%	0%	3%	**22%**	6%	**11%**	0%	6%	8%	3%	0%	8%
Zimbabwe	39	**23%**	8%	0%	3%	**38%**	**10%**	**10%**	0%	0%	0%	0%	3%	5%
**Average for ESA**	**684**	**23%**	**8%**	**2%**	**2%**	**15%**	**9%**	**8%**	**4%**	**2%**	**10%**	**5%**	**3%**	**9%**
**West and Central Africa**														
Benin	42	**17%**	2%	7%	2%	5%	**10%**	5%	0%	7%	**19%**	5%	0%	21%
Cameroon	42	**24%**	0%	7%	0%	**29%**	**17%**	2%	0%	0%	**10%**	2%	0%	10%
Chad	36	**17%**	0%	0%	0%	**14%**	0%	**17%**	0%	0%	6%	**11%**	0%	31%
Congo (Brazzaville)	36	**28%**	0%	6%	0%	**8%**	0%	6%	0%	**11%**	6%	3%	0%	33%
Cote d'Ivoire	39	5%	8%	0%	0%	**10%**	**21%**	**10%**	0%	0%	0%	3%	3%	41%
Gambia	39	**28%**	3%	3%	0%	**18%**	**10%**	0%	3%	5%	**26%**	0%	0%	5%
Ghana	42	**29%**	7%	2%	0%	**12%**	**10%**	**10%**	0%	0%	5%	**12%**	0%	14%
Guinea	39	**31%**	**10%**	0%	3%	**13%**	8%	5%	3%	8%	3%	0%	0%	18%
Guinea Bissau	39	**10%**	**10%**	0%	0%	8%	**51%**	3%	0%	0%	**10%**	0%	0%	8%
Liberia	39	**23%**	**18%**	0%	0%	8%	**18%**	8%	0%	3%	**10%**	5%	0%	8%
Mali	39	**33%**	8%	8%	0%	8%	**10%**	5%	0%	5%	**13%**	5%	0%	5%
Niger	51	**16%**	2%	0%	0%	6%	4%	**14%**	**10%**	2%	**10%**	**10%**	2%	25%
Nigeria	39	**31%**	**10%**	**13%**	3%	**15%**	**13%**	0%	3%	5%	0%	8%	0%	0%
Senegal	33	**27%**	**12%**	3%	0%	6%	6%	0%	3%	3%	0%	9%	0%	30%
Sierra Leone	36	**33%**	3%	3%	0%	6%	**22%**	**11%**	8%	0%	8%	6%	0%	0%
Togo	48	**38%**	0%	4%	0%	**13%**	2%	4%	**13%**	6%	4%	4%	2%	10%
**Average for WCA**	**639**	**24%**	**6%**	**4%**	**<1%**	**11%**	**12%**	**6%**	**3%**	**3%**	**8%**	**5%**	**<1%**	**15%**
**Total across regions**	**1323**	**24%**	**7%**	**3%**	**1%**	**13%**	**11%**	**7%**	**3%**	**3%**	**9%**	**5%**	**2%**	**12%**

* Response to 2023 NCIFP open-ened question: In your opinion, what are the three largest barriers to sustainabiliy for the family planning program in your country?

** No. of respondents in each country multipled by number of responses for 3 barriers; NR is included in the percentages because some respondents listed one or two barriers.

*Enabling environment*

Across the countries, the percentage of responses noting resources, namely reliance on donor financing and issues with domestic financing, as key barriers ranged from a low of 5% in Cote d’Ivoire to a high of 38% in Togo. In 12 of the 17 countries in ESA and in 12 of 16 countries in WCA, resources were the most mentioned barrier. The largest percentages for responses related to resources come from DR Congo and Kenya in ESA (36% each) and Togo in WCA (38%). Respondents in DR Congo (among the 11 of 15 listing resources as the first barrier to sustainability) pointed to “extreme dependence on external funding” affecting programing and “government underfunding, especially for contraceptives.” From Kenya (among the 8 of 12 listing resources as the first barrier to sustainability), one respondent noted “limited resources in a highly competitive resource request environment,” and another described “government laxity to honor FP2030 commitment to fully finance FP commodities by 2026.” Responses in Togo similarly described donor dependence, with one respondent (among the 12 of 16 listing resources as the first barrier to sustainability) citing “the dependence of the program on external financing for the purchase of products and the provision of FP services.”

A range of barriers are included under the theme of political will, policy issues, and accountability. The percentage of responses citing this policy-related theme as a key barrier to sustainability ranged from 0 to 19%. In five countries in ESA and six in WCA, these barriers garnered over 10 percent of responses with the highest percentages in Kenya and Uganda (19% each) and Liberia (18%). In Kenya, one respondent explained that “Lack of political will, inconsistent policies, or changes in leadership can impact the continuity and effectiveness of family planning initiatives.” Three respondents highlighted the policies regarding adolescents and youth, with one noting “lack of policies to give contraceptives to adolescents.” Respondents in Uganda also mentioned restrictions in reaching adolescents and youth, with one citing “restrictive laws for under 18.” Other policy constraints noted included “lack of political will [regarding FP]/conflicting messages from leaders,” and “less prioritization of FP in favor of other country priorities.” In Liberia, respondents highlighted lack of government commitment, with one respondent saying that “lack of leadership and governance in structured program management” hinders sustainability of the FP program.

Nigeria had the highest percentage of responses related to the theme of coordination, planning, and competing interests, noted in 13% of responses (percentages for other countries ranged from 0 to 8%). Respondents noting this as a barrier highlighted lack of follow through on “implementation of planned activities.” One respondent from Nigeria described “dysfunctional health system planning, management and evaluation” as impeding sustainability.

Only a few respondents – in 11 of the 33 countries (8 in ESA and 3 in WCA) – noted private sector engagement as one of the three largest barriers to sustainability. In only two countries (Lesotho and Mozambique) was this barrier included in 5% or more of the total responses for the country. One respondent from Mozambique described “the weak involvement of the private sector and public-private partnerships.” Barriers noted in Lesotho included “private sector exclusion for most commodities,” and “not engaging or collecting data from the private health sector.”

*Program/service delivery*

Human resources, including both numbers of staff and training of staff, were the second most frequently cited as one of the three top barriers to sustainability in most countries. Human resource barriers received between 5 to 38% of responses across the countries and was the most mentioned barrier in three countries: Zimbabwe (38% of responses), Cameroon (29% of responses), and South Africa (28% of responses). In 14 of the 17 countries in ESA, human resources received 10 percent or more of the responses, compared to 9 of 16 countries in WCA. Responses in Cameroon focused on lack of trained providers, with supervision and staff motivation. Responses in Zimbabwe focused on staff attrition, including “brain drain of trained service providers” precipitated by economic conditions in the country. One respondent explained that “training of service providers in FP courses is very expensive for individuals and donor dependence is not sustainable.”

Commodities and supply chain were mentioned as barriers in all but two of the countries across the regions. The percentage of responses identifying commodities and supply chain issues as barriers to sustainability ranged from 0 to 56 percent. More than 10 percent of responses mentioned commodities and supply chain issues in 8 of 17 countries in ESA and 10 of 16 in WCA. While some respondents linked barriers regarding commodities with resource constraints, issues with commodities also included lack of reliable supply and getting products to the last mile, weak forecasting and supply chain management leading to shortages and stockout, issues with local manufacturing, and reliance on import of donor-funded commodities. Among the challenges to sustainability in Guinea-Bissau, the country in which 51 percent of responses focused on commodities and supply chain, were mentions of stockouts of a range of contraceptive methods along with “insufficient logistics.”

Overall, barriers under the theme of infrastructure, coverage, and access comprised an average of 7% of responses, with percentages ranging between 0 and 17 percent in countries. In eight countries in ESA and five in WCA, these barriers were noted by 10 percent or more of respondents. Challenges to sustainability related to infrastructure, coverage, and access, which comprised 17 percent of responses for Chad, included transport, particularly in rural areas, along with insufficient infrastructure and lack of equipment. Access in rural and difficult to reach areas was a common constraint noted across countries. For Rwanda, where 17 percent of responses also relate to infrastructure, coverage and access, respondents noted the role of the Catholic Church in providing health services and the effects on access to a range of contraceptive methods.

In two countries in WCA, over 10 percent of responses centered on quality of care/services. The percentage of responses indicating quality of care/services as among the three top barriers to sustainability ranged from 0 to 13 percent across the countries WCA. For Togo, in which these challenges comprised 13 percent of responses, included poor reception of clients in health centers, lack of information for clients, and lack of client-centeredness in informed and voluntary choice. One respondent in Togo called out the “insufficient user-friendliness of FP services for young people and adolescents.”

In one country in WCA, Congo (Brazzaville), 13 percent of responses noted Data and M&E as among the main three barriers to sustainability. In other countries, 0 to 8 percent of responses highlighted the need for attention to data and M&E. Challenges to M&E and data in Congo (Brazzaville), and in the other 16 countries across the regions that included it, ranged from weak M&E systems, including for monitoring and improving data quality, to low use of evidence for decision-making.

*Attention to socio-cultural context*

Sociocultural, gender and religious barriers were also commonly noted as barriers to sustainability, mentioned in an average of 9 percent of responses across the regions. Across the countries, 0 to 26 percent of responses noted barriers related to sociocultural, gender and religious issues as key challenges to sustainability. For two countries, Benin and Rwanda, the theme of sociocultural, gender and religious barriers received the highest percentage of mentions among the themes. Respondents in Benin, in addition to socio-cultural and normative barriers, noted the low involvement of men with one explaining that “the non-integration of men into the program (husbands who do not always accept supporting the women in adopting the methods),” poses a challenge to sustainability. Socio-cultural, gender, and religious barriers in Rwanda are also apparent in responses for that country: religion and the influence of the Catholic Church looms large among barriers to sustainability according to respondents. Two responses in Rwanda specifically noted the limited access youth have to contraception that is due to cultural barriers. These sociocultural, gender and religious barriers were common themes across the countries.

As a related theme, respondents in some countries also noted that insufficient attention to SBC poses a barrier to sustainability. Although noted in 5 percent of responses overall, in four countries in ESA and three in WCA, 10 percent or more of the responses related to the theme of insufficient attention to SBC as a barrier to sustainability. Respondents in Tanzania, mirroring comments in other countries, noted the myths and misconceptions that need to be addressed in the community, with one noting “bias and myths (both from providers and community),” as barriers to sustainability. Respondents in Chad noted the need to raise awareness about FP and a respondent in Somalia noted the need to foster greater community involvement.

## Discussion

These findings from the 2023 NCIFP and its special questions on Sustainability provide a broad view from 33 counties across two sub-regions of sub-Saharan Africa of expert respondents’ perceptions in that year on the potential for sustainability of the program. The assessments are grounded in expert knowledge of the FP program and highlight the state of sustainability of the program, actions that are being taken, and the range of key challenges their countries face. In addition to asking about the inclusion of FP as part of the country’s plans for UHC, the analysis has addressed issues related to sustainability, grouped under the broad components of donor reliance; coordination among funding mechanisms, data availability from HMIS and LMIS; and planning for sustainability to meet demand (reduce reliance on donors; projection of commodity needs; human resource recruitment and fielding; infrastructure; and subnational support). The components included in the supplement address key elements of the enabling environment and service delivery capacity, with the importance of attention to socio-cultural context emerging in responses to the open-ended question on the three top challenges to sustainability. While the landscape for programs and their sustainability in these countries has drastically changed in 2025, the findings from the 2023 NCIFP provide an important base of evidence.

Both regions scored highest on the extent to which FP is included in plans for UHC, likely due to the global attention to UHC within the Sustainable Development Goals, and its inclusion as target 3.8 under Goal 3 focused on health and wellbeing. Access to reproductive, maternal, newborn and child health is one of four health domains comprising the score for access to UHC (the others are infectious diseases; non-communicable diseases; and service capacity and access) ( United Nations, 2024). Yet, achieving UHC is dependent on strong programming supported with sufficient funding, which has now, with only five years until 2030, become more challenging.

The extent to which governments have developed plans to make their national FP programs more sustainable to meet demand varied, with average scores for each item higher in WCA than ESA. Realistic projections of commodities and programmatic support from leaders at the sub-national level were the highest scoring elements of planning. The relative strength of the scores related to projections of commodity needs is not surprising given the attention to strengthening commodity logistics systems, including forecasting needs, over many decades ( HIP, 2020; USAID Global Health Supply Chain Program, 2017), including through the Reproductive Health Supplies Coalition to which stakeholders from government, private sector and civil society in many of the countries in this paper are members ( https://www.rhsupplies.org/).

The two sustainability supplement questions on reliance on donors (for programs and commodities) stand out as receiving the lowest scores across all of the questions on sustainability, meaning that many FP programs in both SAA regions remain heavily reliant on donor funding - for both commodities and programming. Countries will need to redouble efforts to secure domestic funding. Inclusion of World Bank data on GDP per capita provides some context to the low donor reliance scores, showing that most of the countries with the lowest donor reliance score (heaviest reliance on donor funding) also had the lowest GPDs per capita. These countries are at the highest risk for significant contraction of programs following changes in donor funding, and therefore the least financially sustainable.

While the score related to coordination across funding mechanisms – including national and local government funding, donor financing, and in-kind contributions, among others – received a score of around 60 across the two regions, when asked about plans to make the national FP program more sustainable by reducing reliance on donors, respondents in both regions responded with a middling average score (around 50 in each region), indicating that more work is needed to ensure sufficient domestic resources for the FP program. As participants in the FP2030 Partnership, 22 of the 33 countries included in this paper have made commitments to increase domestic resources for FP, many of those focused on funding for commodities ( FP2030, n.d.).

Findings from the 2023 NCIFP show that some countries such as Tanzania in ESA and Cote d’Ivoire in WCA appear to recognize donor reliance as a threat to sustainability and have developed plans to achieve financial and technical independence, while for others such plans have not yet been created. Planning for staffing recruitment and fielding and for adequate infrastructure were around 50 for ESA and 60 for WCA, indicating heightened attention to planning for these elements of sustainable programming in countries in WCA and room for increased attention to planning for these critical components of program sustainability.

Country-specific analysis of respondents’ perceptions of challenges to sustainability at the country level highlighted commonalities and differences in the prioritization of barriers. While donor dependence and lack of domestic resources top the list of constraints to sustainability in most of the countries, the number of themes that emerged across the enabling environment; program/service delivery; and sociocultural, gender, and religious barriers shows the broad range of challenges respondents who are familiar with the FP program in their countries see the program facing on the path to sustainability.

The percentages of responses within countries gives a snapshot of what barriers are considered most pressing in each country. While domestic financing and donor dependence dominated in all but one country, which other two challenges got the most mentions varied by country. For some countries, commodities and supply chain received more mentions than human resources, and for others infrastructure and access were mentioned more often than infrastructure. In four countries (Burundi, Rwanda, Gambia, South Sudan) over 20 percent of responses focused on sociocultural, gender, and religious barriers affecting sustainability.

Sustainability rests on country-owned and country-managed processes. In addition to identifying funding mechanisms, Chaudhry et al., (2012) highlighted the importance of investing in human and institutional strengthening to cultivate domestic skills for dealing with current and new situations and of strengthening management and stewardship to ensure long-term program sustainability. Experience from other regions shows that attaining sustainability can take years to achieve and that planning along the path to sustainability is important. From this analysis we can see that while there is some progress in planning for sustainability, many of the countries across SSA included in the NCIFP have a ways to go to become sustainable as defined by Khalifa et al., (2001) that “the national family planning program and its public, private and NGO institutions can provide current and potential clients with the information and services necessary to obtain the benefits of quality family planning on a continuous basis without external aid.” Recent shocks to donor funding have added urgency to country sustainability.

### Limitations

This study was conducted in 2023, two years before a seismic shift in donor funding with the abrupt cancellation of the United States government’s support for family planning. Still, the findings provide a country-level snapshot of the programs just prior to this seismic shift. Findings from the countries are based on self-reports from respondents on their perceptions in 2023 of the preparedness of their countries to support sustainable FP programs. Still, the findings represent expert opinion from respondents in each country who are very familiar with the family program and challenges it faces. Furthermore, the internal consistency between responses in the main NCIFP questionnaire and the special questions on sustainability give additional credence to the findings.

While a strength of the sustainability supplement questions is that they were answered in the context of earlier items on the enabling environment for family planning, the number of items that could be added as special questions limited the depth of the analysis possible. The final open-ended question gave respondents the opportunity to state, in their own words, three key challenges to sustainability of their country’s FP program, yet, these brief responses cannot capture the complexity of achieving that state. Still, the range of challenges noted, and the similarities and differences among countries, highlights the importance of context and reinforces the need to resist cookie-cutter approaches to ensuring sustainability. An in depth analysis for individual countries is beyond the scope of this paper, however, we urge others to undertake further analysis. The NCIFP is not designed to measure the impact of programs, for example on contraceptive use, thus further studies on the impact of initiatives to promote sustainability is likewise warranted.

Country context is an important consideration when assessing FP program sustainability. Fiscal space, government effectiveness, and social acceptance of contraceptive use can all impact a program’s ability to offer high-quality services without interruption over the long-term without external support. This study is meant to offer a snapshot of self-reported sustainability scores to summarize a range of country experiences and serve as a starting point for additional analyses. In-depth analysis of individual country contexts contributing to sustainability is beyond the scope of this work.

## Conclusion

The purpose of this analysis was to present the findings from the special questions on sustainability added to the 2023 NCIFP and to provide a broad overview of the challenges a range of countries in SSA face in achieving sustainability – a topic that takes on increased urgency given the current inflection point in donor funding. Further studies to examine the challenges faced by the programs in light of current funding shifts and how they are overcome, or not, would add to our understanding of sustainability of FP programs. This analysis is instructive in highlighting the range of financial and other barriers programs face in achieving sustainability in FP. This paper points to the need for innovative approaches to address common challenges across countries and unique, country-specific, challenges with enabling environment, including financial sustainability; programing and services; and attention to social-cultural, gender, and religious barriers, which affect demand and shape the ability of programs to sustainability meet demand for voluntary and rights-based FP. Countries facing the abrupt funding shifts in 2025 have scant evidence from countries in other regions (see Do et al., 2024) that had more time to plan for donor graduation to help navigate their own swift path to sustainability.

## Ethical approval and consent

Since the first round in 2014, the NCIFP has been conducted within a monitoring and evaluation framework focused on family planning programs, rather than under a research protocol. Still, written informed consent was obtained to take part in the NCIFP and all data has been anonymized.

## Data Availability

Repository name: Zenodo: 2023 National Composite Index for Family Planning (NCIFP): Data File and Questionnaire. https://doi.org/10.5281/zenodo.15058023 This project contains the following underlying data:
• 2023 NCIFP Data File.xlsx (The data file includes the final, cleaned data for the 2023 round of the NCIFP, as well as a codebook identifying the variable names with their corresponding questions. Underlying country data are available from the authors upon reasonable request). • 2023 Questionnaire_English.pdf (The questionnaire is the full questionnaire for the 2023 round of the NCIFP, in English). Data are available under the terms of the Creative Commons Attribution 4.0 International.

## Footnotes

^1^
Attention to socio-cultural context was not included in the close-ended responses, but emerged in the qualitative responses.

^2^
South Sudan has a donor reliance score of 7, indicating high donor dependence, but does not appear in the figure because 2023 GDP per capita data were not available.
